# Preoperative diclofenc reduces postcraniotomy headache: a randomized, placebo-controlled trial

**DOI:** 10.1186/cc10941

**Published:** 2012-03-20

**Authors:** C Molnár, É Simon, J Gál, P Siró, Á Kazup, B Fülesdi

**Affiliations:** 1 University of Debrecen, Hungary

## Introduction

We tested the hypothesis that 100 mg oral preoperative diclofenac reduces postcraniotomy headache.

## Methods

A total of 145 patients having elective craniotonomies were randomly assigned to diclofenac or placebo. Severity of pain was assessed by an independent observer using a visual analogue scale on the day of surgery, on the first postoperative day, and on the fifth postoperative day. The total amount of analgesics administered during the first five postoperative days was converted to intramuscular morphine equivalents. Results were compared using unpaired, two-tailed *t *tests; *P *< 0.05 was considered statistically significant.

## Results

In total, 104 patients had supratentorial and 41 had infratentorial interventions. Sixty-two patients were assigned to placebo and 83 were assigned to diclofenac. The results of VAS scores are shown in Table 1.

**Table 1:** Results of VAS scores

	Placebo	DICLO	*P *value
Day of surgery	4.9 ± 3.5	2.2 ± 3.5	< 0.001
First postoperative day	5.5 ± 3.4	3.7 ± 3.5	< 0.01
Fifth postoperative day	4.3 ± 3.8	2.6 ± 2.9	< 0.01

The relative efficacy of diclofenac was similar in patients having supratentorial and infratentorial surgery. Diclofenac also appeared to be comparably effective in both men and women. Systemic analgesic requirements were reduced during the initial five postoperative days in patients assigned to diclofenac (intramuscular morphine equivalents: placebo = 5.3 ± 4.3 mg vs. diclofenac = 3.6 ± 3.3 mg).

## Conclusion

Preoperatively diclofenac reduces postcraniotomy head-ache compared to placebo, and reduces postoperative analgesic requirements.

